# Tribomechanical Comparison between PVA Hydrogels Obtained Using Different Processing Conditions and Human Cartilage

**DOI:** 10.3390/ma12203413

**Published:** 2019-10-18

**Authors:** Andreia Sofia Oliveira, Oumar Seidi, Nuno Ribeiro, Rogério Colaço, Ana Paula Serro

**Affiliations:** 1 Centro de Química Estrutural (CQE), Instituto Superior Técnico-Universidade de Lisboa, Av. Rovisco Pais 1, 1049-001 Lisboa, Portugalnuno.assuncao.ribeiro@lusiadas.pt (N.R.); 2 Instituto de Engenharia Mecânica Instituto Superior Técnico (IDMEC)-Universidade de Lisboa, Av. Rovisco Pais 1, 1049-001 Lisboa, Portugal; 3 Institut Supérieur des BioSciences (ISBS), École Supérieure d’Ingénieurs de Paris-Est Créteil, 71 Rue Saint-Simon, 94000 Créteil, France; oumar.v.seidi@gmail.com; 4 Departamento de Ortopedia, Hospital Lusíadas Lisboa, R. Abílio Mendes 12, 1500-458 Lisboa, Portugal; 5 Centro de Investigação Interdisciplinar Egas Moniz (CiiEM), Instituto Universitário Egas Moniz, Quinta da Granja, Monte de Caparica, 2829-511 Caparica, Portugal

**Keywords:** poly(vinyl alcohol) hydrogel, freeze-thawing, cast-drying, human cartilage, mechanical properties, tribological behavior

## Abstract

Designing materials for cartilage replacement raises several challenges due to the complexity of the natural tissue and its unique tribomechanical properties. Poly(vinyl alcohol) (PVA) hydrogels have been explored for such purpose since they are biocompatible, present high chemical stability, and their properties may be tailored through different strategies. In this work, the influence of preparation conditions of PVA hydrogels on its morphology, water absorption capacity, thermotropic behavior, mechanical properties, and tribological performance was evaluated and compared with those of human cartilage (HC). The hydrogels were obtained by cast-drying (CD) and freeze-thawing (FT), in various conditions. It was found that the method of preparation of the PVA hydrogels critically affects their microstructure and performance. CD gels presented a denser structure, absorbed less water, were stiffer, dissipated less energy, and withstood higher loads than FT gels. Moreover, they led to friction coefficients against stainless steel comparable with those of HC. Overall, CD hydrogels had a closer performance to natural HC, when compared to FT ones.

## 1. Introduction

Articular cartilage (AC) is a connective tissue whose primary function is to provide a smooth and lubricated joint surface, mediating the transmission of loads with low friction [1]. AC may suffer traumatic degeneration, e.g., as a result of an accident or due to intense sports activity, or progressive degeneration due to loss of structure and function, generally associated with aging or obesity. Because of its avascular nature, cartilage has a poor nutrient supply and a slow turnover of the extracellular matrix, whereby damaged tissues have a limited ability to self-repair [2]. Depending on the nature and extent of damage, regeneration or replacement is possible through different techniques. Procedures such as marrow stimulation (e.g., microfracture, drilling, and abrasion arthroplasty), AC grafts, and cell-based therapies have been successfully used [3,4]. However, these strategies present some disadvantages. For example, stimulation techniques have a limited repairing capacity. In addition, the heat generated by drills and high-speed burrs can cause damage to the subchondral bone [4]. Conversely, autografts cause injury to healthy tissues and provide a limited amount of transplant material, while allografts have the risk of disease transmission and immunogenic reaction, and a higher cost [5]. Recently, tissue engineering has raised interest among the scientific community. The rapid development of molecular biology and transplantation techniques has benefitted the application of mesenchymal stem cells and other cell types in regenerative medicine [6,7,8]. Nevertheless, strong efforts continue to be carried out to develop artificial materials that satisfy the very demanding mechanical and tribological requirements and ensure proper performance as AC substitutes [9].

The characteristics of the biomaterials selected for AC replacement depend on the intended application site, as different loads and ranges of motion can be observed. Most of the actual options for AC substitution use materials that lack the specific architecture and properties of the living tissue, leading to biomechanical failure. Therefore, in the last few years, a substantial effort has been made to mimic the natural cartilage and better approach its mechanical behavior and lubricating mechanism.

In this regard, hydrogels have emerged as promising candidates, since many are biocompatible and present several characteristics comparable to natural tissues, namely the ability to absorb and retain water and other molecules [10]. Some authors explored the possibility of using poly(vinyl alcohol) (PVA) hydrogels due to their biocompatibility, chemical stability, and the possibility of tailoring their properties through different strategies [11,12]. The preparation of PVA gels involves the dissolution of the polymer, followed by crosslinking and eventually further processing techniques [13]. Factors such as the polymer molecular weight, hydrolysis degree and initial concentration, and the method and conditions of crosslinking, or the addition of other compounds, may significantly affect the characteristics of the resulting hydrogel.

Concerning the crosslinking method, chemical and physical approaches have been attempted. Chemical crosslinking implies the use of chemical agents such as borates, glyoxal, aldehydes (glutaraldehyde, acetaldehyde, and formaldehyde) in the presence of methanol, acetic acid, or sulfuric acid, to create acetal bridges between the pendant OH groups of the PVA chains [12,13,14]. The main drawback of using chemicals as crosslinking agents is that they can leave toxic residues, which can compromise the hydrogels’ biocompatibility [12]. Agents like radiation (electron beam or gamma radiation), can also promote the covalent bond between the PVA chains [13,15,16], with the crosslinking density depending on the irradiation dose. Although the irradiation technique does not leave toxic residues, may be used for crosslinking and sterilizing in a single step, and improves cell adhesion [13,14], it is expensive and requires specially trained staff and adequate facilities. Physical crosslinking of PVA hydrogels involves the establishment of non-covalent bonds, i.e., hydrogen bonds between the OH groups of the PVA chains, which leads to the formation of microcrystallites and amorphous zones [11]. It can be achieved through different methods, e.g., cast-drying, freezing-thawing, freeze-drying and theta gelation [11,13,17,18]. Generally, physically prepared PVA hydrogels present a high degree of swelling, an elastic nature (tissue-like viscoelasticity), and higher mechanical strength than those crosslinked by chemical or irradiative techniques [12,13,16,19]. 

Cast-drying (CD) and freezing-thawing (FT) are the most commonly used methods for PVA hydrogels preparation. In the CD method (also called solvent casting or solvent evaporation), the PVA solution (aqueous or not) is slowly evaporated at a given temperature and humidity by natural air drying, hot air drying or freeze-drying [20]. FT induces PVA network reticulation by a combination of three main mechanisms: liquid-liquid phase separation, hydrogen bonding, and crystallite formation [14,21]. During freezing, the growth of ice crystals expels the polymer PVA chains, leading to phase separation and the creation of “rich” and “poor” polymer regions. During the thawing period, voids (pores) appear between the crosslinked regions. The number of FT cycles and the duration of each freezing and thawing step affects the hydrogels microstructure, and consequently, their swelling and mechanical properties.

The main objective of this work was to evaluate the influence of PVA hydrogels preparation conditions on their mechanical and tribological behavior. Hydrogel materials were prepared by CD and FT, in different conditions. After the characterization of their surface morphology, water absorption capacity and thermotropic behavior, mechanical properties were analyzed by compressive tests, and tribological performance was studied through ball-on-disc experiments. The obtained results were compared with those of human cartilage (HC) tested under similar conditions.

## 2. Materials and Methods

### 2.1. Materials

PVA powder (Kuraray Poval^TM^ 28–99) with an average molecular weight of 145,000 ± 21,750 g∙mol^−1^, degree of hydrolysis of 99–99.8 mol% and degree of polymerization of 3300, was used to prepare the hydrogel samples. The pore-forming agents, sodium chloride and sodium bicarbonate were acquired from Sigma-Aldrich (Saint Louis, MO, USA) and PanReac AppliChem (Darmstadt, Germany), respectively. Hydrochloric acid (37% w/w aqueous solution) and phosphate buffered saline (PBS, pH = 7.4 at 25 °C) were both purchased from Sigma-Aldrich. Hyaluronic acid sodium salt (HA) with an average molecular weight of 1–2 million Da and lyophilized bovine serum albumin (BSA, Fraction V, pH 7) used in the preparation of simulated synovial fluid were individually supplied by Carbosynth (Compton, Berkshire, UK) and Serva Electrophoresis GmbH (Heidelberg, Germany). Distilled and deionized (DD) water was obtained using a Milli−Q^®^ Integral 3 water purification system from Millipore (Darmstadt, Germany). 

For the tribological tests, precision balls of austenitic stainless steel AISI 316L, with a diameter of 6 mm, were purchased from Luis Aparicio SL (Barcelona, Spain).

Human articular cartilage (HC) of the femoral condyles was obtained from four distal femurs, harvested from two frozen male cadavers (81 and 82 years old), without any previous lower limb fracture, injury, or reported disease. The material was kept frozen at −80 °C until samples were prepared. The studies with HC were conducted in accordance with the Declaration of Helsinki, and the protocol was approved by the Ethics Committee of Instituto Superior Técnico (Project identification Ref. No. 24/2019 (CE-IST)).

### 2.2. Methods

#### 2.2.1. Materials Preparation

##### Hydrogels

PVA aqueous solutions at 15% w/v were prepared at 95 °C. The solutions were kept at this temperature for 6 h, being gently agitated every hour, to ensure solution homogeneity and avoid gas bubble formation. The resulting polymer solutions were poured into preheated (at 95 °C) glass Petri dishes and then cooled to room temperature for 3 h. 

After that, some PVA solutions were placed at different temperatures (4, 20 and 30 °C) until the weight became constant. The resulting cast-dried (CD) samples were designated CD 4, CD 20, and CD 30 correspondingly (Table 1).

The remaining PVA solutions were processed by the freeze-thawing (FT) method using a different number of cycles and treatment times for the procedures of freezing (at −25 °C) and thawing (approx. 20 °C): 15 cycles of 23 h of freezing and 1 h of thawing for samples FT 23-1 (×15); 15 or 10 cycles of 21 h of freezing and 3 h of thawing for FT 21-3 (×15) and FT 21-3 (×10), respectively; and 5 cycles of 10 h of freezing and 2 h of thawing for FT 10-2 (×5) (Table 1). The Petri dishes containing the PVA solutions were always covered during the freezing time. 

To increase the hydrogels porosity, two approaches were used: (1) following the procedure described by Tai et al. [22], a sodium chloride (SC) aqueous solution (25% w/w) was added to the initial PVA solution in a proportion of 1:10 (v/v), resulting in a ratio of 1:6 (w/w) NaCl:PVA, or (2) sodium bicarbonate (SB) powder was incorporated directly into the initial PVA solution at 5:1 or 8:1 (w/w) NaHCO_3_:PVA ratios, similarly to what was done by Coluccino et al. [23]. Afterward, the PVA-salt solutions were carefully stirred to ensure complete mixing before being transferred to the preheated Petri dishes and submitted to FT under the same conditions used to prepared FT 10-2 (×5) samples, giving rise to FT 10-2 (×5) + SC, FT 10-2 (×5) + SB5 and FT 10-2 (×5) + SB8, respectively (Table 1).

The resulting materials were washed by immersion in DD water for 48 h (replaced three times/day), cut with appropriate dimensions for the characterization tests, and finally stored in the hydrated state in sealed containers until use. The average thickness of the swollen samples was 2.4 ± 0.4 mm.

##### Human Cartilage

Each cadaveric knee was dissected to remove all soft tissue and expose the cartilage surfaces from the distal femur. The femoral condyles were initially sectioned transversely into four cartilage-bone blocks. These blocks were used to obtain cylindrical and rectangular osteochondral plugs, on randomly selected sites. Articular human cartilage (HC) samples were obtained after careful separation of the tissue from the underlying bone. The harvested samples (thickness ≈ 1.9 ± 0.4 mm) were then washed in DD water, carefully blotted in absorbent paper, and stored at −25 °C until ready for use. Prior to each test, prepared specimens were previously hydrated in DD water for 24 h (at 4 °C). 

#### 2.2.2. Materials Characterization

Samples were characterized concerning their surface morphology, water uptake, thermotropic behavior, mechanical properties, and tribological performance. The number of replicates is reported in each case. The quantitative measurements are always given as average ± standard deviation. 

##### Surface Morphology

Discs (diameter 12 mm) of each type of hydrogel were fully hydrated in DD water for 24 h and thereafter, carefully blotted with absorbent paper, placed in a freezer at −80 °C for 30 min and lyophilized (ModulyoD-230, from Thermo Electron Corporation, Asheville, NC, USA) during 48 h. The resulting samples were coated with a thin layer (20 nm) of gold/palladium (Au:Pd = 80:20) using a Quorum Technologies (Q150T-ES) turbo-pumped sputter coater and analyzed in a Field Emission Gun-Scanning Electron Microscope (FEG-SEM, JEOL JSM-7001F, Tokyo, Japan) using an accelerating voltage of 3 kV. For comparison purposes, cylindrical specimens (diameter 12 mm) of HC were also coated with the conductive layer and evaluated. 

##### Water Uptake

Hydrogel discs (8 mm diameter) previously hydrated in DD water for at least 24 h, were dried at 60 °C, and weighed until a constant weight was achieved. The swelling capacity (SC) and equilibrium water content (EWC) were determined using the following equations:

(1) $$\mathrm{SC}\;\left(\%\right)=\frac{W_{\infty }-W_{0}}{W_{0}}\;\times 100,$$

(2) $$\mathrm{EWC}\;\left(\%\right)=\frac{W_{\infty }-W_{0}}{W_{\infty }}\;\times 100,$$

where $W_{\infty }$ is the weight of the swollen hydrogel in equilibrium with water and $W_{0}$ is the weight of the dry hydrogel. The weighing was performed in a semi-micro analytical balance OHAUS (Model Discovery DV215CD). For each tested condition, at least six independent measurements were done. 

The SC and EWC were also determined for HC samples (n = 10, diameter 8 mm) following the same procedure.

##### Thermotropic Behavior

Dry samples with an average weight of 5.8 mg were dried at 60 °C for 8 days. After that, they were sealed in aluminum crucibles and analyzed by differential scanning calorimetry (DSC, 200 F3 Maia, NETZSCH), from 20 °C to 280 °C, at a heating rate of 10 °C∙min^−1^, under a nitrogen gas flow rate of 50 mL∙min^−1^. An empty pan was used as reference. The glass transition temperature (T_g_) was determined as the midpoint in the heat flow baseline change of the endothermic peak, and the melting temperature (T_m_) was considered the middle temperature of the fusion peak. The degree of crystallinity was estimated through the equation:

(3) $$\chi \;\left(\%\right)=\;\frac{∆H}{∆H^{*}}\times 100,$$

where ∆H denotes the heat of fusion, estimated by measuring the area of the peak in the thermograms, and $∆H^{*}$ corresponds to the heat of fusion of a PVA 100% crystalline (138.6 J/g) [24]. 

Hydrated samples with ≈ 5.5 mg were also evaluated by DSC. Samples were cooled to −40 °C and heated to 40 °C at a rate of 10 °C∙min^−1^. The percentage of free/loosely bound (freezable) water present in the swollen materials was determined as:

(4) $$w_{f}\;\left(\%\right)=\frac{∆H_{w}}{∆H_{w}^{*}}\times 100,$$

where $∆H_{w}$ is the enthalpy of fusion of the freezable water, obtained from the total area of the thermogram peaks and $∆H_{w}^{*}$ is the enthalpy of fusion of the pure water (334 J/g [25]). The percentage of freezable water was then subtracted from the EWC (%) to calculate the amount of tightly bound (non-freezable) water ($w_{n}$):

(5) $$w_{n}\left(\%\right)=\mathrm{EWC}\;\left(\%\right)-w_{f}\;\left(\%\right)\;$$

Three independent DSC runs were performed in each case.

##### Mechanical Properties

Uniaxial compression tests were performed in a texturometer TA.XT Express Texture Analyzer (Stable Micro Systems) with a load cell of 50 N in unconfined mode. Sample discs with a diameter of 8 mm, immersed in DD water at 37 °C, were compressed at a strain rate of 0.1 mm∙s^−1^. For each type of hydrogel, at least six samples were tested. The compressive elastic modulus and the elastic and dissipated energies were calculated from the obtained engineering stress-strain curves. HC samples (n = 17, diameter 8 mm) were also assayed at compression for comparison of the biomechanical performance.

##### Tribological Behavior

The friction coefficients (CoF) of PVA hydrogels against 316L stainless steel balls (SS, Ø = 6 mm) were determined in reciprocal oscillating mode using a pin-on-disc tribometer (TRM 1000, Wazau). All experiments were done in lubricated conditions, with phosphate buffered saline (PBS) solution or a simulated synovial fluid (SSF) prepared by dissolving hyaluronic acid (HA, 3 mg∙mL^−1^) and bovine serum albumin (BSA, 4 mg∙mL^−1^) in a PBS solution. The viscosities of the lubricant solutions measured at 20 °C with a viscometer BROOKFIELD DV-II + Pro (AMETEK Brookfield) were 0.83 mPa·s for the PBS solution and 19.93 mPa·s for the SSF solution. Discs of hydrated samples (Ø ≈ 75 mm) were pre-equilibrated for 2 h in the lubricating media before testing. The experiments were done at room temperature (≈20 °C), with a tangential sliding velocity of 43 mm∙s^−1^, a stroke length of 10 mm, and a sliding distance of 13 m. The contact loads were set to 10 and 20 N, which correspond to Hertzian contact pressures of 0.69 and 0.87 MPa, respectively. At least three replicates were performed for each set of conditions.

Friction tests in the same conditions described above were also carried out with HC samples (n ≥ 4, ≈ 2.5 cm length × 2 cm width), using SS balls as counterbody and PBS solution as lubricant.

## 3. Results and Discussion

### 3.1. Surface Morphology

Direct observation of the hydrogels allows us to clearly distinguish between CD and FT hydrogels: while the former are all transparent, the latter present a milky, white appearance. Similar findings are reported by other authors, which attributed it to differences in the network structure, as will be discussed below [11,26,27].

**Figure 1:**
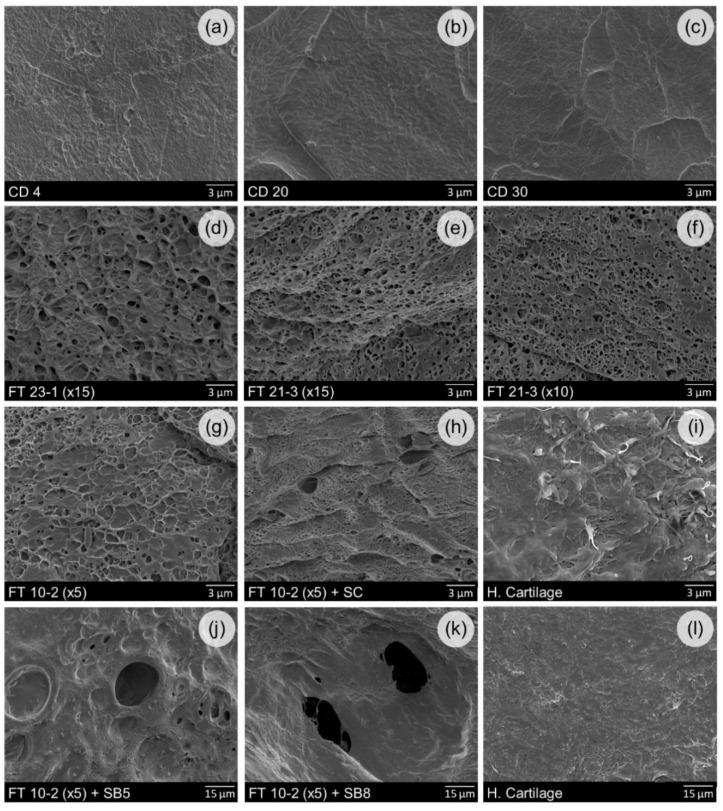
SEM micrographs of PVA hydrogels’ (**a**–**h**,**j**,**k**) and human cartilage samples’ (**i**,**l**) surfaces.

SEM micrographs of the different PVA samples are shown in Figure 1. It can be observed a similarity in the morphology of the materials prepared using the same methodology, i.e., CD or FT (with or without the addition of salts).

CD gels show relatively smooth surfaces with slight differences between the three gelation temperatures (T_gel_ = 4, 20 and 30 °C). Perfetti et al. [28] also did not found significant differences in a study that aimed to evaluate the effect of drying temperature on the morphology of PVA cast films (from room temperature to 70 °C). 

Contrarily to CD materials, FT gels exhibit a porous surface, even in the absence of salts. Suzuki et al. [26] state that PVA hydrogels prepared both by CD and FT consist of swollen amorphous networks that are physically crosslinked by microcrystallites. The nanostructures of both types of hydrogels are similar, with small differences in terms of the average size of the microcrystallites and average distance between them [12,29]. The differences in morphology result from a different distribution of the microcrystallites at the micrometer level: while in FT gels, they are grouped in discrete zones giving rise to crystalline and amorphous regions, on CD gels, they are uniformly distributed [30,31,32].

Porosity is an important characteristic that shall be taken into account in the development of new hydrogels for cartilage replacement. According to Coluccino et al. [23], the introduction of microporosity in the hydrogel contributes to mimicking the natural fluid exudation and pressurization that occurs in the cartilaginous tissue, as well as its load dissipation ability. More, a porous and permeable hydrogel structure will not only facilitate the diffusion of water and other molecules but also cell migration and mechanical anchorage of the implanted material, favoring the in vivo integration [32]. Several techniques can be used to produce interconnected water-based PVA structures with controlled pore size [33,34]. The use of porogens that can be leached out after hydrogel crosslinking [35] has revealed a suitable method, since it allows tuning the pore geometry, by setting the nature, amount and size of the porogen particles.

In this work, the addition of sodium chloride and sodium bicarbonate to the FT gels induced, as expected, the formation of larger pores. A small number of pores (size 1–3 µm) dispersed in an irregular and porous matrix can be observed in the FT 10-2 (×5) + SC samples. For FT 10-2 (×5) + SB5 and FT 10-2 (×5) + SB8 samples, the pores reach average diameters higher than 15 µm. It is known that the porous morphology of PVA gels is affected by both the nature and the concentration of added salts [36]. Patachia et al. [37] observed a large distribution of pore dimensions and shapes in PVA gels prepared with NaCl in the ratio of 1:6 relatively to the PVA weight. In another work, Coluccino et al. [23] demonstrated that it is possible to achieve tunable porosity by mixing different PVA/sodium bicarbonate ratios (from 1:5 to 1:8). They found that the porosity of hydrogels prepared by freeze-thawing increased with the total amount of porogen added, reaching values around 50%, and that the size distribution was not affected by the PVA/porogen ratio.

SEM analysis of HC was performed for comparison purposes. The image presented in Figure 1 shows a dense collagen fiber meshwork on the tissue surface, which is known to be composed of macromolecular units of collagen type II, IX, and XI [38]. Although the tissue seems smooth and intact, with fibers running mainly parallel to the articular surface, a patchy texture can be observed in some locations, with some disassembly of the collagen fibers. This splitting of fibers usually occurs at relatively early stages of osteoarthritis, but it may have been caused during the sample’s preparation. The pattern of collagen fiber meshwork from the articular surface is consistent with previous reports for HC [38,39].

### 3.2. Water Uptake

**Figure 2:**
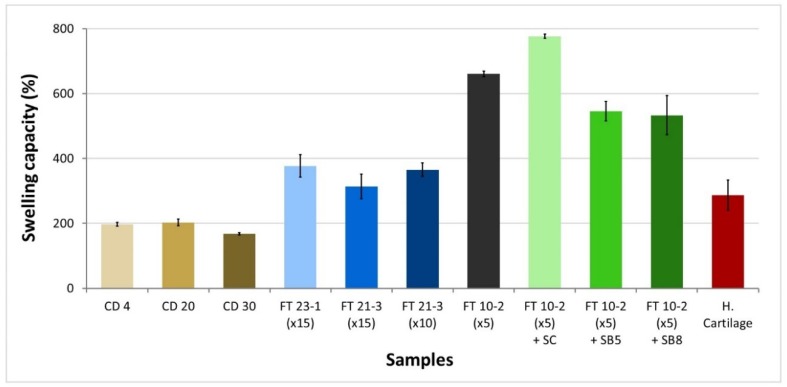
Swelling capacity of PVA hydrogels and human cartilage.

The swelling capacity of the hydrogels was investigated, and the results are shown in Figure 2. It can be observed that CD gels have the lowest swelling ratios, with values that range between 168 and 203%. Among them, CD 30 was the one that absorbed less water. FT hydrogels, prepared without salts, swelled about 1.5–2.2 times more water than CD hydrogels, except FT 10-2 (×5), which swelled more than 3 times. Adding sodium chloride as a pore-forming agent enhanced, even further, the capacity of water absorption, reaching a value of 776%. Sodium bicarbonate originated materials with lower swelling capacities (533%–546%) than observed for the bare reference hydrogel FT 10-2 (×5).

As expected, the most porous hydrogels present higher swelling capacities because of the increased solvent diffusion rate in the matrix. The observed tendency of swelling decreasing, with the increase of drying temperature of CD gels or with the number of freeze-thawing cycles of FT gels, was also observed by Noh et al. [40]. They attribute this to the increased formation of microcrystallites, which is enhanced by reducing the water content (i.e., evaporation of bound water) of polymers with an increase in T_gel_ (in CD gels) or by repeating the FT process (in FT gels). In our study, such tendency on CD gels was only observed for the highest temperature. For lower temperatures, the rate of water evaporation is lower, and therefore, structural differences shall be less pronounced. Concerning FT gels, other authors, like Holloway et al. [41], did not find any relation between the number of freeze-thaw cycles and swelling behavior.

HC has a swelling capacity of about 300%, which is between that observed for CD gels and that of FT gels without salts. Such swelling capacity corresponds to an EWC of about 75% and falls in the range of values found in the literature (EWC = 60%–80% [42]). Swelling of cartilage results from the combined effect of the electrostatic and hydrostatic interactions. It depends on the depth of the tissue (higher values are found near the surface than in the inner zone), decreases with aging, and increases with pathologies like osteoarthritis.

### 3.3. Thermotropic Behavior

**Figure 3:**
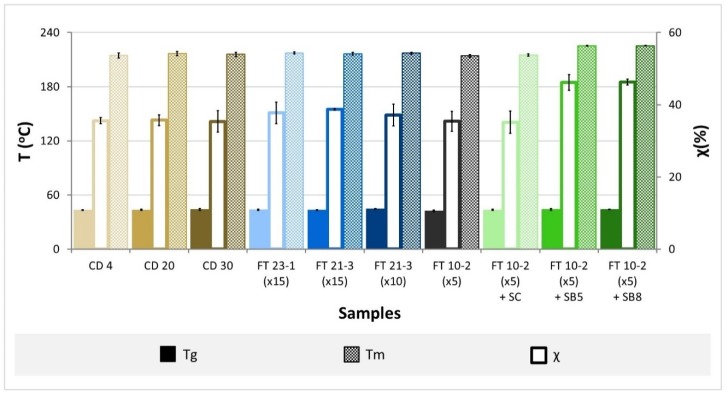
Glass transition temperature (Tg), melting temperature (Tm), and degree of crystallinity (χ) of PVA hydrogels.

DSC thermograms of dry hydrogels are presented in the supplementary information (Figure S1). Two endothermic peaks are shown for all hydrogels. The major peaks correspond to the melting of the polymeric crystallites, while the smaller bulges may possibly be attributed to the glass transition temperature (T_g_), indicating that the polymers have a semi-crystalline structure. The degree of crystallinity estimated from the enthalpy of fusion of the melting peaks using Equation (3) is given in Figure 3, together with the glass transition (T_g_) and melting (T_m_) temperatures, which were obtained for all samples.

The glass transition temperatures of all samples fall in a straight range of temperatures, 43.2–44.6 °C, showing that the degree of crosslinking of the hydrogels is similar. Gupta et al. [43] found close values (40–42.5 °C) for PVA materials prepared by freeze-thawing with 8–14% of the polymer. Higher values (70.5–73.4 °C) were observed by Nkhwa et al. [44] for FT gels obtained from solutions with 10%–30% PVA. For CD hydrogels prepared with the same PVA concentrations (10%–30%), they verified that T_g_ decreased from 73.4 to 58 °C when the polymer concentration increased.

Concerning the melting temperatures, they also presented similar values for the different studied hydrogels, all falling in the range of 214.2–217.3 °C, except for the gels prepared with sodium bicarbonate, where higher values were observed (225.2–225.4 °C). The obtained results are in the range of those found by other authors (210–215 °C [44]), although higher temperatures are also common (e.g., 220.6–227 °C [45]), depending on the characteristics of the PVA used to prepare the hydrogels.

The estimated degrees of crystallinity assume values between 35.2 and 38.7% for all the gels and higher for FT 10-2 (×5) + SB5 and FT 10-2 (×5) + SB8 samples (around 46.2%), probably due to the presence of the salt.

**Figure 4:**
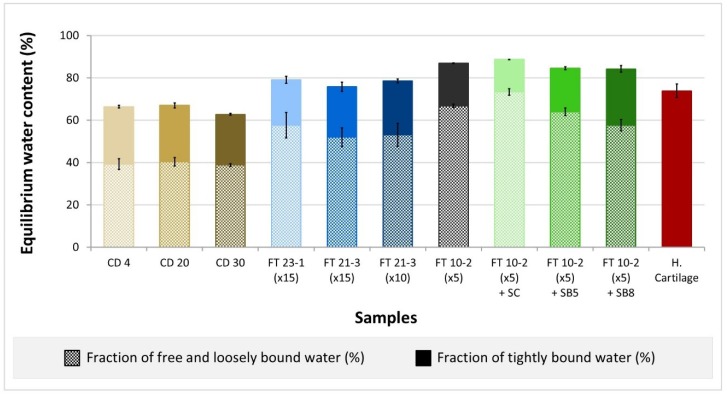
Equilibrium water content (EWC) values of PVA hydrogels and human cartilage. Fractions of free/loosely bound water and tightly bound water contained in the PVA samples.

DSC thermograms of the swollen hydrogels are also provided in the supplementary information (Figure S2). They present two overlapping endothermic peaks between −20 and 20 °C, which may be attributed to the fusion of freezable water that interacts in two different ways with the polymeric matrix [45]: loosely bound water, weakly associated with the polymer and with T_f_ < 0 °C, and free water that does not interact with the polymer and presents T_f_ ≥ 0 °C. Tightly bound water, considered non-freezable, corresponds to water molecules directly bound to the polar groups of the polymer matrix through hydrogen bonds and is not detected in the obtained thermograms since it only freezes at T_f_ < −93 °C. The fractions of free/loosely bound water and tightly bound water contained in the studied PVA gels are shown in Figure 4 (data reported in Table S1 in the supplementary information).

In all cases, the fraction of freezable water (free/loosely bound) is higher than that of non-freezable water (tightly bound), assuming values of 59%–83% of the total water content. FT samples absorb more water than CD samples and present a higher fraction of freezable water. In global terms, the comparison of water content obtained for the PVA gels (63% to 87%) with that of HC, tested in the same conditions (75%), shows that they are relatively close and in agreement with other values reported in the literature (typically 60%–80% [43]). It should be stressed that the water content is an important property since it affects the material’s behavior e.g., permeability, strength, and elasticity [43].

### 3.4. Mechanical Properties

**Figure 5:**
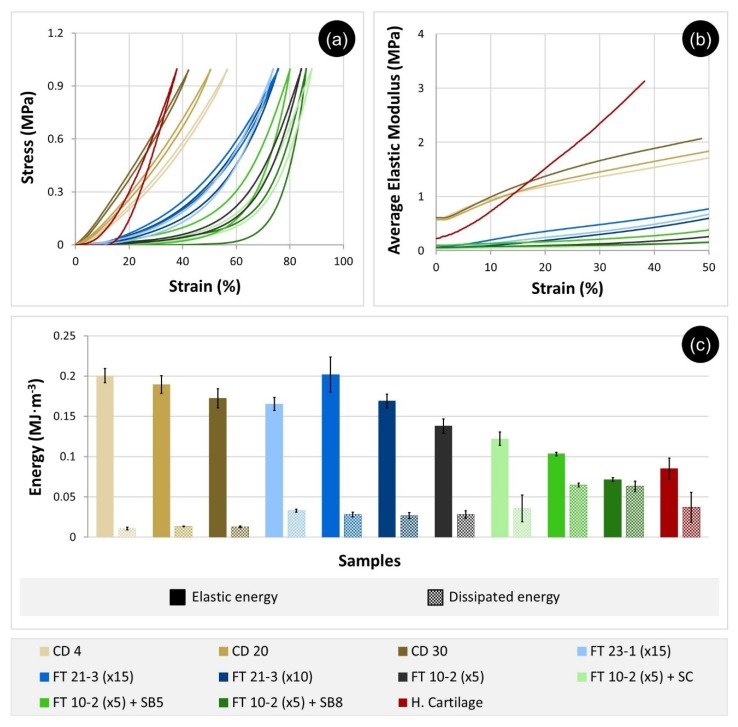
Typical compression stress-strain curves (**a**), elastic modulus variation (**b**), and elastic and dissipated energies (**c**) of PVA samples and human cartilage.

The stress-strain curves of the compression tests performed with PVA hydrogels are shown in Figure 5a. The different groups of samples reveal a similar mechanical response to compression loads within each group. While CD samples exhibited an almost linear behavior, FT samples led to curves with a variable slope, with the existence of two distinct zones being evident, especially when the samples were prepared with salts. CD gels also contrasted with FT ones in their viscoelastic behavior: FT samples showed higher hysteresis and, consequently, dissipated more energy.

The variation of the elastic modulus (slope of the stress-stain curves during loading) with the strain (up to 50%) and the elastic and dissipated energies (area of the hysteresis loop and total area under the unloading curve, respectively) were calculated from the acquired data and are displayed in Figure 5b,c. The results reveal that among the studied hydrogels, CD present the higher elastic modulus for a given strain and dissipate less energy (≈0.01 MJ∙m^−3^). FT gels prepared with 10 or 15 cycles and without the addition of salts have elastic modulus significantly lower than those found for CD samples, elastic energies within the same range of those gels, and higher dissipated energy (≈0.03 MJ∙m^−3^). The curves of the FT gels produced within 5 cycles demonstrate that, independently of the addition of pore-forming agents, these materials are the ones with the lowest elastic modulus, highest dissipated energies (0.03–0.06 MJ∙m^−3^), and lowest elastic energies (0.07 to 0.14 MJ∙m^−3^). 

Murakami et al. [46] also compared the mechanical properties of PVA materials obtained by CD and FT and found that the elastic modulus of CD gel was significantly superior to that of FT gel. This behavior shall be related to the lower porosity of CD materials, which present a more compact structure and lower water content. Coluccino et al. [23] produced PVA hydrogels with different porosities by adding sodium bicarbonate and observed a decrease in the compressive modulus with the increase of porosity. The results show that, although porosity is an important characteristic to mimic the natural cartilage and favor the bio-integration of the synthetic materials, it impairs the mechanical performance of the materials.

A comparison of the stress-strain curves of the hydrogels with HC shows that the CD gels are the ones that present the closest behavior to the natural tissue. HC can be seen as a biphasic 3D network with hierarchical structural characteristics, that shows both creep and stress relaxation behaviors [47]. Its viscoelastic properties are determined by the way the fluid flows through the extracellular matrix. Cartilage exhibits a nonlinear permeability response when submitted to compression, which constitutes a protective mechanism for the tissue. In fact, increasing the pressure and strain enhances fluid exudation from the cartilage and its stiffening, which prevents more fluid from flowing. The results presented in Figure 5b confirm this behavior. The mechanical properties of cartilage depend on the type of cartilage and its location since collagen fibers’ organization varies from zone to zone. According to the literature, the elastic modulus in compression may assume values that range from 0.13 to 1.91 MPa [48], depending not only on the type and location of the cartilaginous tissue but also on the measurement conditions. It shall be stressed that, because of its unique structure, cartilage exhibits anisotropic properties, and its stiffness in tension is generally 5–20 times higher than in compression [48].

### 3.5. Tribological Behavior

**Figure 6:**
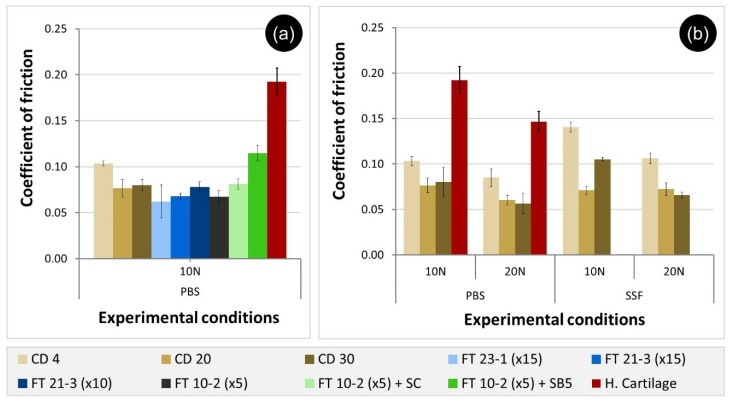
Friction coefficients measured for the tribological sliding pairs hydrogel/SS 316L and human cartilage/SS 316L under 10 N of load using PBS as lubricant (**a**) and for CD hydrogels/SS 316L and human cartilage/SS 316L under 10 and 20 N of load using both PBS and SSF solutions as lubricants (**b**).

Figure 6a shows the average CoF values for PVA hydrogels tested under 10 N of loading in PBS. The majority of the hydrogel samples exhibit CoF values in the range 0.062 to 0.081. However, CD 4 and FT 10-2 (×5) + SB5 presented significantly higher friction values: 0.103 ± 0.003 and 0.115 ± 0.008, respectively. FT 10-2 (×5) + SB8 sample did not withstand the applied load (10 N), and for that reason, no values for CoF could be measured. Tests carried out with HC in the same conditions led to a higher value of CoF (0.192 ± 0.015), which shows that all hydrogels, except FT 10-2 (×5) + SB8, reveal promising tribological properties at this load.

Comparison with data available in the literature is not straightforward since study conditions are not standardized, and the CoF values are strongly affected by the system geometry and configuration, test parameters (e.g., sliding speed, applied load) and lubricant, among other factors. However, the values obtained for the PVA hydrogels are within the range found by other authors: Pan et al. [49] tested FT gels against SS balls in different media (distilled water, physiological saline, and bovine serum), under loads between 5 and 15 N and sliding speeds 45–225 rpm and found CoF values in the range 0.03–0.075. Also, Kosukegawa et al. [50] got values between 0.01 and 0.1 for the CoF of PVA gels tested against SS balls under different pressures (0.022–0.146 MPa) and sliding speeds (0.5–600 mm·s^−1^). Agreement with literature data was also observed for cartilage assay. For example, Li et al. [51] measured the CoF of HC pins against SS plates in Ringer’s solution, with a contact pressure of 0.4 MPa and a sliding speed of 2 mm·s^−1^, and reached values of 0.266.

An attempt to use a load of 20 N was also performed with all the FT materials, but these did not resist and cracked during the assay. Therefore, for this normal force, only the CoF for the CD materials were obtained (Figure 6b). Overall, as the normal force increased, a tendency to decrease the friction values was observed. The same was observed with HC. Both hydrogel and HC are permeable to interstitial fluid and deform during friction tests. According to Amontons’ First Law, the CoF should be directly proportional to the normal force. However, several authors verified that both hydrogels and cartilage do not obey this law, and instead, the CoF depends on stiffness, roughness, speed, and load [52,53].

In the experiments carried out in SSF, equal or higher CoF values were registered with both applied forces. Due to the increased viscosity of the fluid (19.93 mPa·s), which contains albumin and hyaluronic acid, it was expected that the thickness of the lubricating layer between the contact surfaces would increase, thereby reducing the friction between the sliding pairs. The higher CoF values may be attributed to the interactions between the biomolecules present in the lubricant and the matrix of the PVA gels. Indeed, both the initial conformation of the adsorbed molecules and eventual structural changes that occur during the experiment are responsible for the observed behavior [54]. In recent work, Nečas et al. [53], also found that protein free-lubricants led to lower CoF than solutions containing albumin and/or γ-globulin, in tests performed with a metal/UHMWPE sliding pair. They concluded that friction is dependent on both protein type and concentration in the lubricant, as well as on the kinematic conditions. Depending on the sliding velocity, the protein molecules may partially or totally denature and lose their original secondary structure, leading to adsorbed protective films with different thicknesses and viscoelastic properties. 

Nevertheless, further tribological studies at higher sliding distances should be performed with the systems studied here to evaluate the evolution of both CoF and wear rates in these materials.

## 4. Conclusions

PVA hydrogels are materials with a high potential to be used in cartilage replacement since they can be tailored to exhibit properties similar to the native biological tissue. The preparation conditions of such materials are critical for their performance. In this work, different strategies were followed to produce such hydrogels: cast-drying (CD) and freeze-thawing (FT). 

CD gels presented a compact structure and a relatively smooth surface. Contrarily, FT gels showed some porosity, which could be increased through the addition of salts that acted as pore-forming agents. As a consequence of their non-porous structure, the CD gels exhibited a lower water absorption capacity. The differences in the production method almost did not affect the materials’ thermotropic properties. The crystallinity estimated from the DSC measurements was similar for all hydrogels. FT gels presented a higher fraction of freezable water (free/loosely bound) than CD gels. Concerning mechanical properties, gels showed similar behavior when using the same production methodology. CD gels were stiffer and dissipated less energy. In friction tests, carried out against SS in PBS solution, similar values of CoF were obtained for the majority of the studied hydrogels. However, FT samples revealed a lower wear resistance. Due to its superior performance, CD gels were selected to be tested under a higher load and in the presence of biological molecules (albumin and hyaluronic acid). The increase in load generally led to lower values of CoF, while the biomolecules maintained or increased the CoF. 

For comparison, HC was tested in the same conditions of the studied hydrogels. Overall, CD gels were the ones that presented more similarities with the natural tissue in terms of morphology, water content, and mechanical behavior, and also led to lower CoF values. Thus, when cartilage repair is no longer possible, CD gels seem to be more suitable and promising as potential substitutes for articular cartilage.

## Figures and Tables

**Table 1:** Conditions of preparation of the cast-dried and freeze-thawed samples.

Cast-Drying		Freeze-Thawing				
Samples	Drying Temperature (°C)	Samples	Freezing Time/Cycle (h)	Thawing Time/Cycle (h)	Number of Cycles	Pore-Forming Agent
CD 4	4					
CD 20	20					
CD 30	30					
		FT 23-1 (×15)	23	1	15	-
		FT 21-3 (×15)	21	3	15	-
		FT 21-3 (×10)	21	3	10	-
		FT 10-2 (×5)	10	2	5	-
		FT 10-2 (×5) + SC	10	2	5	NaCl (1:6 w/w) *
		FT 10-2 (×5) + SB5	10	2	5	NaHCO_3_ (5:1 w/w) *
		FT 10-2 (×5) + SB8	10	2	5	NaHCO_3_ (8:1 w/w) *

* Ratios relative to PVA weight.

## Supplementary Materials

The following are available online at https://www.mdpi.com/1996-1944/12/20/3413/s1, Figure S1: DSC thermograms of dry PVA hydrogels, Figure S2: DSC thermograms of hydrated PVA hydrogels, Table S1: Estimated percentage values of free/loosely bound and tightly bound water contained in the hydrogels.
