# Supplementary material for: Tribomechanical Comparison between PVA Hydrogels Obtained Using Different Processing Conditions and Human Cartilage
Source: Materials (Basel). 2019 Oct 18;12(20):3413. doi: 10.3390/ma12203413 (PMC6829290; doi:10.3390/ma12203413)
Supplement: Supplementary file 1 [file materials-12-03413-s001.pdf]

materials

MDPI

Article

Tribomechanical Comparison between PVA Hydrogels Obtained Using Different Processing Conditions and Human Cartilage

Andreia Sofia Oliveira [1,2], Oumar Seidi [3], Nuno Ribeiro [1,2,4], Rogério Colaço [2,*] and Ana Paula Serro [1,5,*]

1 Centro de Química Estrutural (CQE), Instituto Superior Técnico-Universidade de Lisboa, Av. Rovisco Pais 1, 1049-001 Lisboa, Portugal; andreia.oliveira@tecnico.ulisboa.pt (A.S.O.); nuno.assuncao.ribeiro@lusiadas.pt (N.R.)
2 Instituto de Engenharia Mecânica Instituto Superior Técnico (IDMEC)-Universidade de Lisboa, Av. Rovisco Pais 1, 1049-001 Lisboa, Portugal
3 Institut Supérieur des BioSciences (ISBS), École Supérieure d'Ingénieurs de Paris-Est Créteil, 71 Rue Saint-Simon, 94000 Créteil, France; oumar.v.seidi@gmail.com (O.S.)
4 Departamento de Ortopedia, Hospital Lusíadas Lisboa, R. Abílio Mendes 12, 1500-458 Lisboa, Portugal
5 Centro de Investigação Interdisciplinar Egas Moniz (CiiEM), Instituto Universitário Egas Moniz, Quinta da Granja, Monte de Caparica, 2829-511 Caparica, Portugal
* Correspondence: rogerio.colaco@tecnico.ulisboa.pt (R.C.); anapaula.serro@tecnico.ulisboa.pt (A.P.S.); Tel.: +351-218-419-240 (A.P.S.)

Supplementary information

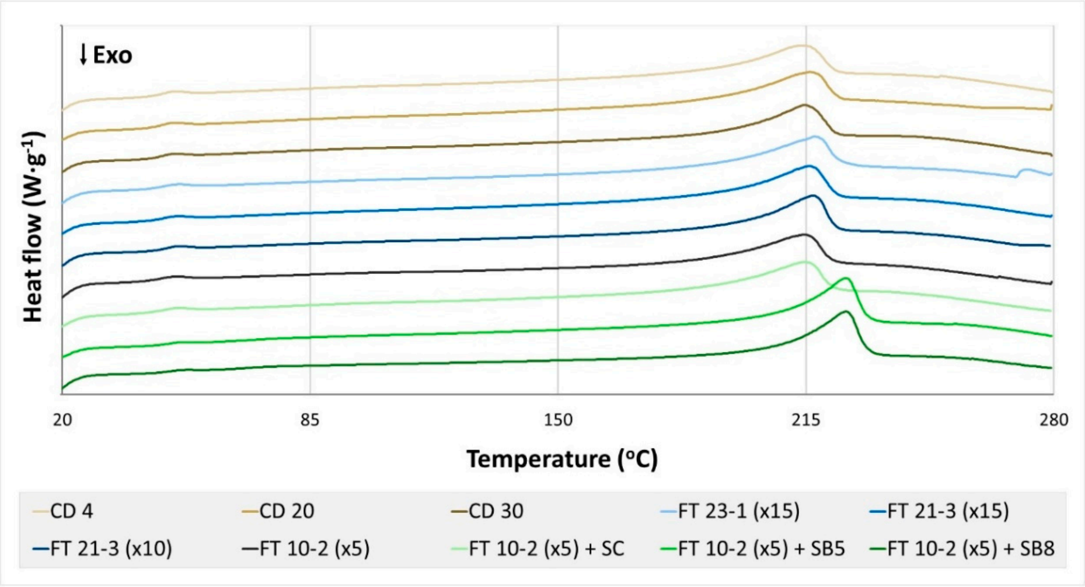

Figure S1. DSC thermograms of dry PVA hydrogels.

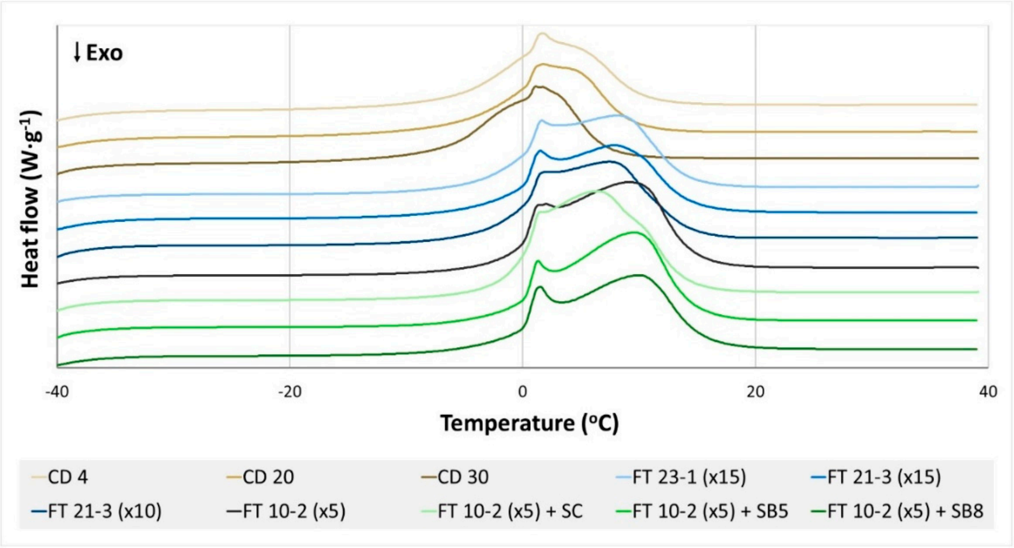

Figure S2. DSC thermograms of hydrated PVA hydrogels.

Table S1. Estimated percentage values of free/loosely bound and tightly bound water contained in the hydrogels.

Samples	ΔH ($J·g^{-1}$ of Hydrated Sample)	Equilibrium Water Content (%)	Free/Loosely Bound Water (%)	Tightly Bound Water (%)
**CD 4**	130.8 ± 8.4	66.4 ± 0.6	39.2 ± 2.5	27.2 ± 2.5
**CD 20**	134.5 ± 6.8	66.9 ± 1.2	40 ± 2	27 ± 2
**CD 30**	129.7 ± 2.1	62.7 ± 0.4	38.8 ± 0.6	23.9 ± 0.6
**FT 23-1 (×15)**	192 ± 20	78.9 ± 1.6	57 ± 6	21 ± 6
**FT 21-3 (×15)**	173.2 ± 15.1	75.7 ± 2.2	51.9 ± 4.5	23.8 ± 4.5
**FT 21-3 (×10)**	176.8 ± 18.3	78 ± 1	52.9 ± 5.5	25.5 ± 5.5
**FT 10-2 (×5)**	222.9 ± 2.5	86.9 ± 0.1	66.7 ± 0.7	20.1 ± 0.7
**FT 10-2 (×5) + SC**	244.9 ± 5.2	88.6 ± 0.1	73.3 ± 1.6	15.3 ± 1.6
**FT 10-2 (×5) + SB5**	213.6 ± 6.1	84.5 ± 0.7	64.0 ± 1.8	20.5 ± 1.8
**FT 10-2 (×5) + SB8**	192.4 ± 9.1	84.1 ± 1.6	57.6 ± 2.7	26.5 ± 2.7
